# Therapeutic effects of progesterone and its metabolites in traumatic brain injury may involve non-classical signaling mechanisms

**DOI:** 10.3389/fnins.2013.00108

**Published:** 2013-06-13

**Authors:** Paul S. Cooke, Manjunatha K. Nanjappa, Zhihui Yang, Kevin K. W. Wang

**Affiliations:** ^1^Department of Physiological Sciences, College of Veterinary Medicine, University of FloridaGainesville, FL, USA; ^2^Departments of Psychiatry and Neuroscience, Center for Neuroproteomics and Biomarker Research, McKnight Brain Institute, University of FloridaGainesville, FL, USA

**Keywords:** progesterone receptor, membrane receptors, allopregnanolone, progesterone receptor membrane component 1, controlled cortical impact

## Abstract

Traumatic brain injury (TBI) is an important and costly medical problem for which no clinically proven treatment currently exists. Studies in rodents and humans have shown beneficial effects of progesterone (P4) on both mortality and functional outcomes following TBI. Neuroprotective effects of P4 in TBI likely involve the classical nuclear progesterone receptors (Pgr) that are widely distributed in both glial cells and neurons of the brain. However, P4 may have critical effects not mediated through Pgr. In the brain, P4 is converted to a metabolite, allopregnanolone (ALLO), whose beneficial effects equal or exceed those of P4 in TBI. ALLO does not bind Pgr, suggesting it acts through non-classical pathways. ALLO has effects on GABA_A_ and pregnane X receptors, as well as on the mitochondrial permeability transition pore. In addition, ALLO is metabolized to another compound, 5alpha-dihydroprogesterone, which binds Pgr, suggesting ALLO actions may involve signaling through Pgr as well as the aforementioned mechanisms of action. P4 and ALLO also signal through a number of membrane receptors (progesterone receptor membrane component 1, and membrane progesterone receptors (mPRs) alpha, beta, gamma, delta, and epsilon) in the brain that are distinct from Pgr, although the role of these receptors in the normal brain and in the therapeutic response to P4 and ALLO following TBI is unclear. In summary, P4 has the potential to become the first clinically effective treatment for TBI, and the effects of P4 and its metabolite ALLO in TBI may involve Pgr, mPRs, and other signaling pathways. Elucidating these mechanisms will more clearly reveal the potential of classical and non-classical pathways to mediate important effects of P4 and its metabolites, and potentially offer new therapeutic approaches to TBI.

## Introduction

Traumatic brain injury (TBI) is defined as a neurotrauma resulting from a mechanical force, such as that caused by rapid acceleration or deceleration, blast waves, crush, an impact, or penetration by a projectile. TBI is a major cause of death and disability worldwide, especially in children and young adults. The Centers for Disease Control and Prevention (CDC) estimate that there are approximately 1.4–2.0 million incidents each year in the U.S. involving TBI. Of these, nearly 100,000 patients die, another 500,000 are hospitalized, and thousands of others suffer short- and long-term disabilities. Leading causes of TBI in the U.S include violence, transportation accidents, construction, and sports. Falls account for the greatest number of TBI cases (34%), with motor vehicle crashes and traffic-related incidents (17%), struck by/against events (16%), and events and assaults (10%) also being important contributors (Faul et al., 2010). CDC estimates of TBI do not include injuries seen at U.S. Veterans Health Administration Hospitals. Firearms and blast injuries from explosions are major causes of TBI. Among the 1.6 million American warfighters returning from Iraq and Afghanistan, between 5 and 35% sustained a mild TBI or concussion during their deployment, with 80% being caused by blast exposures (Champion et al., 2009).

A wide variety of treatments have been and are currently used for TBI, including diuretics such as mannitol to reduce intracranial pressure, hypothermia and medically induced coma. In cases where intracranial pressure remains high for an extended period of time, surgical interventions such as decompressive craniectomy are commonly used (Maas et al., 2008). However, although all of these methods may have potential benefits, and in some cases are widely used, no treatment has been shown to have clear benefits in any of the large number of randomized clinical trials that have been conducted (Maas et al., 2008). Therefore, despite the prevalence of TBI, there presently are no clinically proven effective treatments.

Progesterone (P4) and its metabolites have shown great promise as potential treatments for TBI. Progesterone is an ovarian steroid hormone that regulates many facets of reproduction (Lydon et al., 1995; Conneely et al., 2001). Over the past two decades, an increasing body of literature (reviewed in Sayeed and Stein, 2009; Stein and Wright, 2010; Stein, 2012) has documented the remarkable beneficial effects of P4 on mortality and morbidity following TBI. These treatments were originally developed using animal models that replicate the various aspects of human TBI pathophysiology, such as the controlled cortical impact injury model utilized extensively in rats and mice. More recent results have indicated that this treatment may show equal promise in human patients following TBI (Sayeed and Stein, 2009; Stein and Wright, 2010; Stein, 2012).

The initial presumption is that beneficial P4 effects following TBI are mediated through classical nuclear progesterone receptors (Pgr). However, identification of several membrane progesterone receptors (mPR) that are ubiquitously expressed in the brain (Meffre et al., 2005; Intlekofer and Petersen, 2011; Pang et al., 2013) and the demonstration that allopregnanolone (ALLO), a P4 metabolite that does not bind Pgr, had effects in TBI models equaling or exceeding those of P4 [reviewed in Sayeed et al. (2009)] calls this into question. Here we review current understanding of the mechanism of action of P4 and metabolites in TBI, with an emphasis on non-Pgr mediated pathways. Complete elucidation of how P4 and related compounds work could present new possibilities for improving P4 therapy or developing second-generation treatments for TBI.

### Progesterone production and function in brain

The term neurosteroid was coined by Baulieu in 1981 to describe steroids produced in the brain as a result of de novo synthesis or metabolism of steroid precursors from the peripheral circulation that alter the activity of neurons or other cells types (Baulieu, 1981). Progesterone is a neurosteroid, and is produced in both glial and neuronal cells of the CNS (Baulieu et al., 2001).

Nuclear Pgr, like other steroid hormone receptor superfamily members, is a ligand-activated transcription factor and is widely distributed throughout female reproductive organs. Reproductive and non-reproductive organs in males also express Pgr (Shughrue et al., 1988). In both sexes, Pgr concentrations are high in hypothalamic regions regulating reproductive cyclicity, and occur throughout the brain of both mature and developing animals. Almost all brain areas express Pgr, although clear regionalities in relative amounts are observed (Intlekofer and Petersen, 2011). Various cell types, including neurons, glia, and other cell types (reviewed in Brinton et al., 2008) express Pgr, indicating that most brain cells are potential P4 targets.

The exact role of P4 signaling in non-reproductive brain areas is unclear. Actions of P4 and metabolites in the brain are pleiotropic, and its effects on Schwann cell proliferation and myelination, memory, cognition, neural excitability, glial cell function, inflammation, neurogenesis and neural progenitor cell proliferation are well-known (Brinton et al., 2008). In addition to its extensive roles in brain, P4 effects on pathologies as diverse as Alzheimer's disease, stroke and post-traumatic stress disorder, as well as TBI, have been documented or suggested (Brinton et al., 2008).

## Progesterone effects in animal models of TBI

Work using animal models has shown that P4 and its metabolite ALLO produce a remarkable array of beneficial short- and long-term effects following TBI (Table 1). The neuroprotective role of P4 was first reported by Stein and colleagues (Roof et al., 1992, 1993), who showed that female rats recovered better than males did following TBI. Subsequent hormone replacement experiments indicated that P4 was similarly neuroprotective in males and females following TBI (Roof et al., 1994). These initial findings were confirmed and expanded by many different groups using a variety of TBI models (Sayeed and Stein, 2009; Stein and Wright, 2010; Stein, 2012; Table 1). Progesterone and its metabolite ALLO are effective in reducing the brain swelling and edema that are TBI hallmarks, and P4 has beneficial effects on blood brain barrier function and intracranial pressure following TBI (Ishrat et al., 2010). P4 ameliorates the increases in inflammatory cytokines such as TGF-β, TNF-α, IL-6, and IL-1β that occur after TBI (He et al., 2004a,b), decreases gliosis and apoptosis (Djebaili et al., 2004, 2005; He et al., 2004b), and increases expression of anti-oxidant enzymes such as superoxide dismutase (Pajović et al., 1996). Most critically, the beneficial effects occurring in the first few days following TBI are also accompanied by significant improvements in long-term deficits in cognition and functionality normally experienced by animals after TBI (Djebaili et al., 2004, 2005; Espinoza and Wright, 2011; Stein, 2012). For example, P4 administration to rats following TBI led to improved locomotor activity and performance on the Morris water maze test used to assess learning and spatial memory (Djebaili et al., 2004, 2005; He et al., 2004a,b). More recently, P4 has been reported to decrease anxiety following TBI (Djebaili et al., 2005). Overall, these studies clearly showed that P4 improved cognitive and behavioral recovery, the primary goal of any neuroprotective agent.

**Table 1:** **Representative effects of progesterone (P4) and allopregnanolone (ALLO) treatment following traumatic brain injury**.

**Treatment**	**Species**	**Effect**	**References**
*P4, ALLO*	Rat	Decrease in inflammatory cytokines (e.g., IL-Iβ, TNF-α)	He et al., 2004a; VanLandingham et al., 2006; Anderson et al., 2011
*P4, ALLO*	Rat, Mouse	Improved spatial learning performance in Morris Water Maze	Roof et al., 1994; Goss et al., 2003; Djebaili et al., 2004; He et al., 2004a; Jones et al., 2005; Roof et al., 2005
*P4, ALLO*	Rat	Decreased apoptosis and neuronal death	Roof et al., 1992; Djebaili et al., 2004; He et al., 2004a; VanLandingham et al., 2006; Anderson et al., 2011
*P4, ALLO*	Rat	Decreased reactive gliosis	Goss et al., 2003; Djebaili et al., 2005; VanLandingham et al., 2006
*P4, ALLO*	Rat	Decreased edema	Roof et al., 1992; VanLandingham et al., 2006; Kasturi and Stein, 2009
*P4*	Rat	Decreased impairment of blood-brain barrier	Cutler et al., 2007; Ishrat et al., 2010
*P4*	Rat	Decreased expression of proapoptotic proteins	Djebaili et al., 2005
*ALLO*	Human, Rat	Increased proliferation of neural progenitor cells	Wang et al., 2005
*P4, ALLO*	Rat	Decreased anxiety following TBI	Cutler et al., 2006; Baykara et al., 2013
*ALLO*	Rat	Decreased mitochondrial damage	Kaasik et al., 2003; Sayeed et al., 2009
*P4*	Rat	Increased expression of brain-derived neurotrophic factor (BDNF)	Cutler et al., 2006
*P4, ALLO*	Rat	Increased signaling through GABA_A_ receptors, with decreased neuronal excitotoxicity	He et al., 2004a
*P4*	Rat	Decreased intestinal inflammatory response	Chen et al., 2008
*P4*	Human	Decreased mortality, improved functional outcomes	Wright et al., 2007; Xiao et al., 2008

## Progesterone effects in human clinical trials

The success of initial animal studies led to human clinical trials to determine if P4 could produce similar benefits in humans. Progesterone was shown to be neuroprotective for humans following TBI in two small-scale pilot studies and is presently being tested in more extensive phase III clinical trials for moderate to severe TBI (Stein, 2012). A pilot study funded by NIH called Progesterone for Traumatic Brain Injury, Experimental Clinical Treatment (ProTECT) was a 3-year, randomized, double-blind, placebo-controlled phase 1 and 2 trial, with a total of 100 adult TBI patients. Progesterone was found to be safe and produced no adverse events in the participants. Among 77 patients receiving progesterone and 23 receiving placebos, P4 reduced the overall death rate by 50% compared with placebo, with significant improvements in functional outcomes and level of disability among patients with brain injury (Wright et al., 2007; Table 1). A concurrent trial of P4 in China utilizing 159 TBI patients also showed that P4 significantly lowered the mortality rate and produced improved functional outcomes (Xiao et al., 2008; Table 1).

These promising results led to the design and initiation of ProTECT III, a currently on-going NIH-funded phase III clinical trial evaluating P4 for TBI treatment in a larger patient population (ProTECT III, 2009). This national study began enrolling patients in 2009 and is a multicenter, randomized, double-blind study involving 1,140 patients over 5 years at 17 U.S. medical centers. Another phase III trial (1180 patients) sponsored by BHR Pharma LLC (the SyNAPSe trial) is also underway to evaluate the effectiveness of a proprietary progesterone formulation in treating severe TBI.

## Are critical effects of progesterone and ALLO in TBI mediated by non-classical signaling pathways?

Actions of P4 in reproduction are mediated in large part by the classical nuclear receptor, Pgr, as shown by infertility and loss of most P4 functions in female progesterone receptor knockout (PRKO) mice lacking nuclear Pgr (Lydon et al., 1995; Conneely et al., 2001; Jeong et al., 2005). Despite the centrality of Pgr to P4 function, some studies suggest effects of P4 in the brain might not be solely due to binding to Pgr.

Ciriza et al. (2006) showed that the synthetic progestin medroxyprogesterone acetate, which has high affinity for Pgr and high potency as a progestin for most endpoints, was not neuroprotective. Conversely, VanLandingham et al (VanLandingham et al., 2006) demonstrated that an enantiomer of P4 did not activate Pgr-mediated transcription but still had neuroprotective effects.

**Figure 1:**
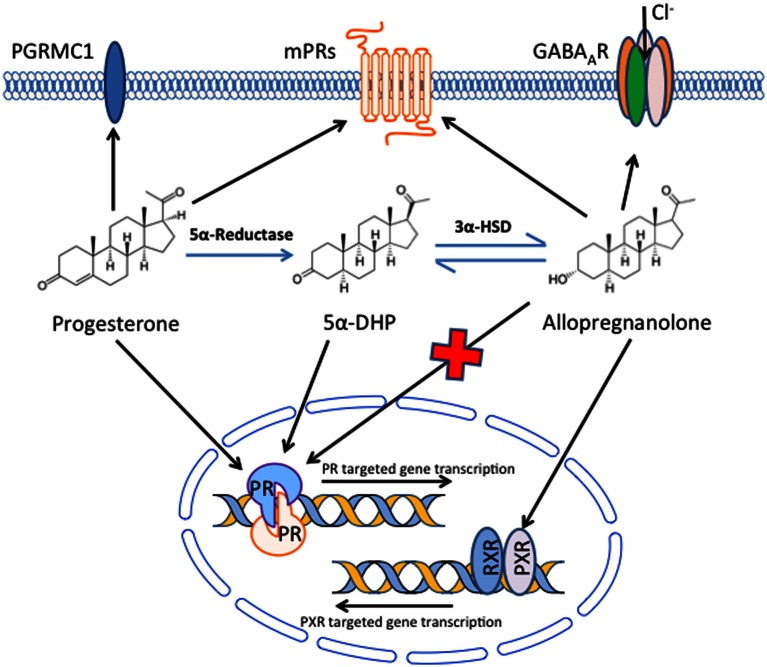
**Potential mechanisms by which progesterone and its metabolites signal in the brain to produce beneficial effects following traumatic brain injury**. Progesterone, either from the systemic circulation or produced locally in the brain, can bind and signal through the classical nuclear progesterone receptors, but also binds receptors in the plasma membrane, including PGRMC1 and a family (α–ε) of membrane progesterone receptors (mPRs). In addition, progesterone can be converted to 5α-dihydroprogesterone (5α-DHP) and then allopregnanolone in the brain. Allopregnanolone can bind and alter ion flux through GABA_A_ receptors, and also binds pregnane X receptors (PXR), although the native progesterone does not bind GABA_A_ receptors and has limited affinity for PXR. In addition, although allopregnanolone does not bind to nuclear progesterone receptors, it can be converted to 5α-DHP, which does have affinity for nuclear progesterone receptor. Allopregnanolone also binds to mPRs, although its affinity for PGRMC1 has not been reported. Thus, a variety of nuclear and membrane receptors may be involved in the beneficial effects of progesterone and its metabolites following traumatic brain injury.

Several lines of evidence have suggested that beneficial effects of P4 in TBI may be partially or even entirely due to metabolites of P4 produced in the brain, rather than the parent molecule itself. Extensive data indicates that the major brain metabolite of P4, ALLO, may be signaling in large part through pathways other than Pgr and that membrane receptors may be significant factors in mediating therapeutic effects of both P4 and ALLO (Figure 1).

Allopregnanolone is a metabolite of P4 that is produced in the brain (Baulieu, 1981; Baulieu et al., 2001) and may have critical roles as a neurosteroid in both normal brain and in therapeutic responses to P4 treatment after TBI (Table 1). Allopregnanolone is produced from P4 by the sequential action of two steroidogenic enzymes (Figure 1). Progesterone is first converted to 5α-dihydroprogesterone (5α-DHP) by type 1 5α-reductase. The 5α-DHP is then further converted to ALLO by 3β-hydroxysteroid dehydrogenase (3β-HSD) (Nin et al., 2011; Wang, 2011). Both 5α-reductase and 3β-HSD are expressed at highest levels in neurons, with minimal expression in glial cells (Agís-Balboa et al., 2006), suggesting that ALLO production is at least predominately from neurons, although ALLO could have paracrine actions on other brain cells. Critically, Ciriza et al (Ciriza et al., 2006) demonstrated that inhibiting normal metabolism of P4 to 5α-DHP and ALLO abolished the neuroprotective effects of P4. These results suggest that metabolites of P4 may play key roles in neuroprotective effects of P4, and that the mechanism of action of these compounds may not be exclusively through nuclear Pgr.

Extensive work demonstrated that ALLO is highly neuroprotective in TBI animal models, with beneficial effects equaling or exceeding those obtained with P4 (Table 1). Administration of ALLO to rats following TBI reduced the increase in inflammatory cytokines and apoptotic cell death in the brain and the functional deficits in cognition and memory typically seen following TBI (Djebaili et al., 2004, 2005; He et al., 2004a,b).

Critically, ALLO does not bind to the nuclear Pgr (Figure 1) that is the major target of P4 (Rupprecht et al., 1993), suggesting that ALLO could have independent actions differing from those of P4. The ALLO generated by conversion of P4 to ALLO (Figure 1) can be partially metabolized back to 5α-DHP *in vivo*, and this 5α-DHP binds nuclear Pgr and induces transcriptional changes typical of P4 (Rupprecht et al., 1993). Based on its ability to be metabolized to 5α-DHP, it seems possible that ALLO actions in TBI are at least predominately mediated through nuclear Pgr.

Recent groundbreaking work by Liu et al. (2012) has suggested, however, that neuroprotective effects of ALLO may be independent of Pgr. This group used PRKO mice to test the neuroprotective effects of P4 in a stroke model. They reported that ALLO had beneficial neuroprotective effects in PRKO mice subjected to experimental stroke, while P4 was ineffective. These results emphasize that Pgr is obligatory for protective effects of P4 in stroke. Importantly, these results also indicate that ALLO actions can occur in mice lacking Pgr, strongly supporting the idea that beneficial ALLO actions are predominately independent of Pgr (Liu et al., 2012).

## Mechanism of ALLO actions in TBI

If ALLO activity is independent of Pgr, determining how ALLO acts could potentially have significance for clinical TBI treatment. A variety of mechanisms have been suggested, including binding to GABA_A_ receptors, mPRs, and nuclear receptors such as pregnane X receptor (PXR).

## Effects through γ-amino butyric acid type A (GABA_A_) receptors

The GABA_A_ receptor is a ligand-gated ion channel. Upon binding to ligands such as GABA, the GABA_A_ receptor selectively conducts chloride ion into cells, causing hyperpolarization that inhibits neurotransmission. GABA_A_ receptors are expressed in both neurons and glial cells distributed throughout the brain (Pirker et al., 2000). ALLO at nanomolar concentrations binds to GABA_A_ receptors and potentiates the action of endogenous GABA, whereas at micromolar concentrations ALLO directly activates GABA_A_ receptors (Figure 1), and thus may be able to reduce neuronal death following TBI or other insults (Paul and Purdy, 1992; Lambert et al., 2001; Magnaghi et al., 2006). For example, P4 and ALLO protect cerebellar Purkinje cells *in vitro* and *in vivo* from ischemia (Ardeshiri et al., 2006; Kelley et al., 2008, 2011). Furthermore, P4 effects were mediated by ALLO acting through GABA_A_ receptors: treatment with finasteride, a 5α-reductase inhibitor that prevents metabolism of P4 to ALLO, abolished the protective effects of P4 while administration of a GABA_A_ receptor antagonist abolished neuroprotective effects of both P4 and ALLO (Ardeshiri et al., 2006).

ALLO stimulates neural progenitor proliferation (Wang et al., 2005; Wang and Brinton, 2008) and neurogenesis of cerebellar granule cells (Keller et al., 2004) and decreases neuronal cell death, and all of these effects appear to be through GABA_A_ receptors. Interestingly, ALLO showed similar neuroprotective effects through activation of GABA_A_ receptors in PRKO and wild-type mice after spinal cord injury (Labombarda et al., 2013). Furthermore, ALLO has anxiolytic, anticonvulsant and anesthetic properties believed to be mediated through GABA_A_ receptors and potentially involved in its effects on TBI. These effects do not require Pgr, because ALLO effects on these endpoints were similar in wild-type and PRKO mice (Reddy et al., 2004, 2005; Reddy and Zeng, 2007).

## ALLO effects mediated through PXR

The PXR is a promiscuous receptor activated by diverse group of endogenous or exogenous compounds, including steroids (Lamba et al., 2004; Ma et al., 2008). PXR is expressed in various parts of the brain and spinal cord (Lamba et al., 2004; Mellon et al., 2008), including human cerebral microvessel endothelial cells that form the blood brain barrier (Chan et al., 2011).

ALLO activates mouse PXR *in vivo* and *in vitro* (Figure 1) and induces PXR target genes (Lamba et al., 2004; Langmade et al., 2006). PXR activation is known to mediate anti-inflammatory activities in the intestine (Mencarelli et al., 2010) and anti-apoptotic activities in the liver (Zucchini et al., 2005). Neuroprotective effects of ALLO after TBI involving anti-inflammatory, anti-apoptotic effects or other actions may be partially mediated through PXR in brain or other organs. The use of brain-specific or global PXR knockout mice in TBI studies will clarify the role of PXR in neuroprotective effects of ALLO after TBI.

A variety of other ALLO targets in TBI have also been proposed, including the mitochondrial permeability transition pore (MPTP). The MPTP plays a major role in initiating necrosis or apoptosis in the brain following TBI (Crompton, 1999), and neuroprotective effects of ALLO may be partially mediated through stabilization of MPTP (Kaasik et al., 2003; Sayeed et al., 2009). Understanding interactions between ALLO and MPTP will be essential for completely elucidating its mechanism of action and determining whether ALLO can have significant beneficial effects in TBI that are additive or partially additive to those seen with P4.

### Role of membrane progesterone receptors (mPRs) in therapeutic effects of P4 and ALLO

Several mPRs have been described, and some evidence suggests these could be involved in therapeutic effects of P4 and/or ALLO following TBI (Figure 1). Progesterone receptor membrane component 1 (PGRMC1), originally cloned in 1996 from porcine liver (Falkenstein et al., 1996), is widely expressed in brain (Meffre et al., 2005; Guennoun et al., 2008; Intlekofer and Petersen, 2011) and increased following brain injury (Guennoun et al., 2008). PGRMC1 is expressed in regions involved in cerebrospinal fluid production and osmoregulation, suggesting it could be involved in beneficial effects of P4 on the blood-brain barrier and osmoregulation following TBI (Meffre et al., 2005).

PGRMC1 is expressed in the plasma membrane and other cellular locations (Falkenstein et al., 1999; Sakamoto et al., 2004; Peluso, 2006). Progesterone and other steroids bind to PGRMC1 in membrane fractions (Falkenstein et al., 1999), but whether P4 actually binds to and signals through PGRMC1 has been questioned (Cahill, 2007; Price, 2013). ALLO binding to PGRMC1 has not been analyzed. Interestingly, PGRMC1 protein levels were up-regulated in neurons and were induced at the site of injury in astrocytes after TBI (Meffre et al., 2005). It has been hypothesized that P4 protects the brain after injury by up-regulating brain derived neurotrophic factor (BDNF), a neurotrophin known to promote survival of neurons and play a role in cognition, anxiety-like behavior and pain (Gonzalez et al., 2005). In glial cells, P4 acts through PGRMC1 to increase BDNF (Su et al., 2012). Furthermore, a role for PGRMC1 in synaptogenesis of developing Purkinje cells in neonatal rats has been suggested (Sakamoto et al., 2004). Treatment with P4 after spinal cord injury up-regulates PGRMC1 without affecting Pgr expression, and this neuroprotective role of P4 acting through PGRMC1 could also occur in brain following TBI (Labombarda et al., 2003; Guennoun et al., 2008).

Increased proliferation of neuroprogenitor cells from adult rat hippocampus induced by P4 is mediated through PGRMC1 because these cells lack Pgr and proliferation was prevented by inhibiting PGRMC1 mRNA (Liu et al., 2009). Various reports suggest PGRMC1 might mediate P4 effects on axonal regeneration and growth (Runko and Kaprielian, 2004; Sakamoto et al., 2004) and promote survival of neuronal cells (Gonzalez et al., 2005) by preventing apoptosis after TBI. Studies using global and brain-specific PGRMC1 knockout mice will be useful to establish the role of PGRMC1 in P4 and ALLO effects.

A number of other mPRs have been described that are structurally distinct from PGRMC1, but have significant homology with each other. These mPRs were discovered in fish oocytes and then later reported in other vertebrates including humans (Zhu et al., 2003a,b). Five mPR subtypes have been characterized: mPRα, mPRβ, and mPRγ are coupled to inhibitory G-proteins and inhibit intracellular cAMP generation while mPRδ and mPRε are coupled to stimulatory G proteins and increase intracellular cAMP (Pang et al., 2013). P4 and its metabolites including ALLO bind mPRs and initiate signaling (Thomas and Pang, 2012).

Expression of mPRs is extensive in the rat, mouse and human brain (Intlekofer and Petersen, 2011; Zuloaga et al., 2012; Meffre et al., 2013; Pang et al., 2013), but the role of these mPRs (if any) in mediating protective effects of P4 and ALLO after TBI is unclear. Among the mPRs, mPRα is the most studied and mediates numerous P4 effects in fish (Zhu et al., 2003b; Tubbs and Thomas, 2009; Dressing et al., 2010), but also has effects on human breast cancer cells (Dressing et al., 2012) and GnRH release from rodent GnRH neurons (Sleiter et al., 2009). In rodents, mPRα was localized in the neurons, but not the glia, of normal animals. However, mPRα expression was induced in glial cells (oligodendrocytes, astrocytes, and reactive microglia) after TBI. These observations led the authors to conclude that mPRα may play a role in mediating neuroprotective effects of P4 (Zuloaga et al., 2012). Furthermore, P4 and ALLO prevented apoptosis and death of serum-deprived immortalized GnRH secreting neurons that express high levels of mPRs (α, β, δ, ε) but negligible levels of Pgr (Thomas and Pang, 2012). Recently, P4 and ALLO were reported to inhibit starvation-induced cell death and apoptosis in rat hippocampal neuronal cells that show higher levels mPRδ and mPRε mRNAs compared to mPRα and mPRβ and relatively low Pgr expression (Pang et al., 2013). These results suggest possible neuroprotective roles of mPRs in mediating P4 and ALLO effects after TBI, but whether mPRs are involved in neuroprotective effects of P4 and ALLO, as well as the potential specific roles of the various mPR subtypes, remains to be established.

In summary, protective roles of P4 and ALLO may involve signaling through a number of non-classical receptors, in addition to Pgr. Elucidating these mechanisms of action will advance our understanding of the roles of P4 and its metabolites in normal brain function, as well as shed light on the therapeutic possibilities of these compounds for treatment of TBI and other neurodegenerative diseases.

### Conflict of interest statement

The authors declare that the research was conducted in the absence of any commercial or financial relationships that could be construed as a potential conflict of interest.

## Abbreviations

ALLO: allopregnanolone (3α-hydroxy-5α-pregnan-20-one)
CDC: Centers for Disease Control and Prevention
5α-DHP: 5α-dihydroprogesterone
3β-HSD: 3β-hydroxysteroid dehydrogenase
GABA: γ-amino butyric acid
mPR: membrane progesterone receptor
MPTP: mitochondrial permeability transition pore
PXR: pregnane X receptor
P4: progesterone
PRKO: progesterone receptor knockout
Pgr: progesterone receptor
PGRMC1: progesterone receptor membrane component 1
ProTECT: Progesterone for the Traumatic Brain Injury, Experimental Clinical Treatment
TBI: traumatic brain injury.
